# Chemical Composition and Antioxidant, Antiviral, Antifungal, Antibacterial and Anticancer Potentials of *Opuntia* *ficus-indica* Seed Oil

**DOI:** 10.3390/molecules27175453

**Published:** 2022-08-25

**Authors:** Abeer S. Alqurashi, Luluah M. Al Masoudi, Hamida Hamdi, Abeer Abu Zaid

**Affiliations:** 1 Department of Biology, Faculty of Sciences, Taif University, P.O. Box 11099, Taif 21944, Saudi Arabia; 2 Zoology Department, Faculty of Science, Cairo University, Giza 12613, Egypt; 3 Department of Food Science and Nutrition, Alkhurmah University College, Taif University, Taif 21974, Saudi Arabia

**Keywords:** *Opuntia ficus-indica*, fixed oils, antioxidant, antifungal, antibacterial, antiviral, cytotoxicity

## Abstract

*Opuntia ficus-indica* (OFI) is a cactus that is widely cultivated in the Kingdom of Saudi Arabia especially in the Taif region due to its favorable weather for growing, and it has benefits as a food and traditional medicine. The aim of the current study was to chemically characterize *Opuntia ficus-indica* seed oil from Taif, Kingdom of Saudi Arabia, using GC-MS and HPLC analysis and evaluate its antioxidant, antiviral, antifungal, antibacterial and anticancer activities. Linolenic acid was the dominating fatty acid in OFI oil, followed by oleic acid, linoleic acid, palmitic acid and stearic acid. Total tocopherol (α-, β-, Ɣ-tocopherol) was found to be 24.02 μg/mL. Campesterol was the main phytosterol, followed by γ- & β -sitosterol, and Stigmasterol. The phenolic components scored 30.5 mg gallic acid equivalent per ml of oil with 89.2% antioxidant activity (% DPPH radical inhibition) at 200 µL/mL of OFI oil. OFI oil showed an inhibition efficacy against microbial strains especially *Saccharomyces cervisiae* with a diameter (28.3 ± 0.4), MBC (15 µg/mL) and MIC bacteriostatic (10 µg/mL). While OFI oil had no effect against *Aspergillus niger*, OFI oil showed weak inhibitory activity against A-2780 (Ovarian carcinoma) cell line, although it showed significant inhibitory activity against PC-3 (Prostate carcinoma) cell line. OFI oil exhibited an antiviral effect (22.67 ± 2.79%) at 300 µg/mL of Oil against herpes simplex type 2 (HSV-2) virus. The bioactive compounds of OFI oil, as well as its main biological activities, make it a promising candidate for the non-communicable disease management.

## 1. Introduction

Botanical medicines are widely used due to their reliable efficacy, reduced side effects and relative economic cost [1]. Nowadays, the demand for nutrients, natural components and health-boosting foods is permanently increasing [2,3]. There are about 2,253 medicinal plants in various regions of the Kingdom of Saudi Arabia [4,5]. *Opuntia ficus-indica* (OFI) of the family Cactaceae, known as prickly pear, comprises about 1500 species [6]. It was originally grown in different regions of Saudi Arabia especially in the City of Taif for the edible prickly pear fruit and consumed by local populations as an important food source [7]. Moreover, the OFI plant is widely spread in South America, Australia, South Africa and the Mediterranean area [8]. It is a tropical or subtropical plant up to five meters high with a thick, woody stem [9].

Various studies have shown that prickly pear seed oil is edible, and with potential importance to the agriculture industry [10]. Many researchers have been interested in studying the phytochemical profile of the seed oils of two Opuntia species *O. ficus-indica* and *O.dillenii* and have found that the two seed oils are rich in very active molecules, such as unsaturated fatty acids, sterols, tocopherols and polyphenols [3,11].

The highest benefit of this oil due to its high amount of polyunsaturated fatty acids, especially linoleic and linolenic acids, which have potential health avails due to their roles as the eicosanoids biosynthesis precursors [3,12]. Oil extraction by cold screw pressing is an alternative method and has been found to be a substitute to extraction of solvent [13]. This process has the advantage of being less oil producing than others, but is safer, simpler, less expensive, hygienic, no chemical residue and ecologically friendly [14]. Cold pressed oils improve the quality of oil and rich with bioactive components such as essential fatty acids, sterols, tocopherol, and phenolics [15].

To our knowledge, there are few studies about *Opuntia ficus-indica* seed oil growing in Saudi Arabia. Therefore, the aim of current study was to chemically characterize *Opuntia ficus-indica* seed oil growing in Saudi Arabia using GC-MS and HPLC analysis and evaluate its antioxidant, antiviral, antifungal, antibacterial and anticancer activities.

## 2. Materials and Methods

### 2.1. Oil Extraction

Seeds of *Opuntia ficus-indica* were collected from Taif City. Natural oil 100% extracted by cold pressing using a screw extractor in a local maesarat, Taif, Saudi Arabia. Finally, the oil was stored at 20 °C until analysis.

### 2.2. Identification of Opuntia ficus-indica Seed Oil

#### 2.2.1. Fatty Acid Composition

Fatty acid analysis was carried out in triplicate, consisting of two successive steps, fatty acid methyl ester (FAME) preparation and chromatographic analysis. Lipids-extract esterification was performed according to the method of [16]. Determination of fatty acid methyl esters was performed by comparing their retention times with pure standards. Their quantification according to their percentage are taken out by the peak integration. Data were expressed as individual fatty acids percentages in the lipid fraction.

#### 2.2.2. Sterols and the Various Components

Gas chromatography/mass spectrophotometer was used for the identification and quantification of sterols and the various components of *Opuntia ficus-indica* oil. Sterols were converted to trimethylsilyl (TMS) ether derivatives prior to analysis by gas chromatography [17]. Sterols were analyzed as their TMS ethers by capillary gas chromatography with flame ionization detection. The GC parameters were as previously described [17]. Identification of sterols and various components was based on relative retention times of commercially-available compounds, comparison with literature data [18,19] and mass spectral analyses (NIST/EPA/NIH 1999). Quantitative data were calculated by comparing the average peak area of the component to the total areas.

#### 2.2.3. Tocopherols

Tocopherol Analysis was performed by HPLC-(Agilent 1100), consisting of two LC- pumps and a UV/Vis detector with a C18 column (125 mm × 4.60 mm, 5 µm particle size). Agilent ChemStation is used to analyze the obtained Chromatograms. Conditions of Chromatography were as previously described [20].

#### 2.2.4. Determination of Total Phenolic Compounds

Total phenolic compounds (TPC) in *Opuntia ficus-indica* seed oil were determined spectrophotometrically according to the colorimetric method of Folin–Ciocalteu [21]. Data expressed as mg gallic acid equivalent (GAE) per ml of oil.

### 2.3. Antioxidant Activity by Free Radical Scavenging Assay:

The free radical scavenging activity was estimated using 1,1-diphenyl-2-picrylhydrazyl (DPPH) radical as illustrated by [22]. The positive control was BHT. Results expressed as % of inhibition of the DPPH radical (Equation (1)). The IC_50_ is equivalent to 50% of DPPH inhibition.

(1) $$\%\;of\;inhibition\;of\;the\;DPPH\;radical=\left[\frac{Abs_{control}-\left(Abs_{sample}-Abs_{blank}\right)}{Abs_{control}}\right]\times 100$$

*Abs_control_* = The DPPH absorbance

*Abs_sample_* = The sample absorbance

*Abs_blank_* = The ethanol negative control absorbance

### 2.4. Antimicrobial Activity of Opuntia ficus-indica Seed Oil

#### 2.4.1. Microbial Strains

The OFI oil’s antimicrobial activity was determined versus seven pathogenic microorganisms mentioned in the following: Gram-positive bacterial strains (*Staphylococcus aureus*, *Bacillus subtilis*); Gram-negative strains (*Escherichia coli*, and *Klebsiella pneumoniae*); a strain of yeast (*Saccharomyces cerevisiae*) and fungi (*Aspergillus niger*, *Penicillium digitatum*). All pathogenic isolates were obtained from department of microbiological laboratories, Faculty of science, Cairo University, after its isolation and identification. Oxytetracycline (OT30) and penicillin (P10) were used as positive control [23,24].

#### 2.4.2. Antimicrobial Activity

Disk diffusion agar method was used to determine the antibacterial and antifungal activities of OFI oil [25]. Microdilution assay for bacterial strains by using sterile Mueller–Hinton media and for antifungal tests potato dextrose agar (Scharlab, S.L, Barcelona, Spain) was performed. Cell suspensions (0.1 mL) of bacterial strains were adjusted to 10^8^ CFU/mL Cell Forming Units and 10^5^ spores/mL for fungus by MacFarland, and then inoculated onto the surface of agar plates. Then, sterile discs were made (3 mm in diameter) into inoculated plates, and 25 µL of oil filled into each disc. Dishes were placed for 2 h to allow the oil to diffuse and incubated at 37 °C for 48 h for yeast, 24 h for bacterial strains, and 3–4 days for fungi. The negative control was carried out without oils. Antimicrobial activity was calculated by measuring the area of inhibition zone around the discs. Strains tests were replicated three times.

#### 2.4.3. Evaluation of MIC and MBC

The broth dilution method was used to determine Minimum Inhibitory Concentration (MIC) of OFI oil against microorganisms. Pre-modified 0.01mL microbial strains were inoculated into tubes containing. 50.0, 45.0, 40.0, 35.0, 30.0, 25.0, 20.0, 15.0, 10.0, 5.0 µL/mL OFI oil and incubated 24 h at 37 °C. The results were evaluated by showing visible growth inhibition of microbial tubes (no turbidity). The Minimum Bactericidal Concentration (MBC) was introduced by subculture, in which partition of the ~10 µL of each tube invisible growth used in MIC onto Mueller–Hinton agar medium and at 37 °C, 24 h. Colony growth was examined, and all tests were repeated three times.

### 2.5. Cytotoxic Assay of Opuntia ficus-indica Seed Oil

#### 2.5.1. Mammalian Cell Line

Vero cell (derived from the kidney of African green monkey), PC-3 cell line (Prostate carcinoma), and A2780 cell line (Ovarian carcinoma) were purchased from the American Type Culture Collection (ATCC, Manassas, VA, USA). The cells were cultured in Dulbecco’s modified Eagle’s medium (DMEM) boosted with 10% heat-inactivated fetal bovine serum, 1% l-glutamine, buffer of HEPES and 50 µg/mL gentamycin. The cells were maintained at 37 °C in a humidified 5% CO_2_ atmosphere and were subcultured twice a week [26].

#### 2.5.2. Cytotoxicity Evaluation

The cytotoxicity of fixed oil against each cell lines (Vero, PC-3 and A-2780) were determined through MTT colorimetric method by [27]. The 50% inhibitory concentration (IC_50_), the concentration demanded to produce toxic effects in 50% of healthy cells, was determined.

#### 2.5.3. Antiviral Evaluation

The cytopathogenic herpes simplex type (2HSV-2) virus was propagated and assayed in confluent Vero cells [28]. Spearman–Karber method was used to enumerate infectious viruses by determining the tissue culture infectious dose 50% (TCID_50_) with eight wells per dilution and 20 µL of inoculum per well [29].

##### Antiviral Activity

The cytopathic effect inhibition assay was used to estimate the antiviral screening. This assay will be chosen to demonstrate specific inhibition of a biological function, and the MTT method was used to measure a cytopathic effect in sensitive mammalian cells [30,31]. Acyclovir was used as positive control in this assay.

### 2.6. Statistical Analysis

Statistical analysis of data was carried out using GraphPad Prism 5. The data were analyzed for statistical significance by the one-way analysis of variance, followed by Tukey’s multiple comparison tests. The data represented as mean ± standard error (*SE*). Data at *p* < 0.05 were considered significant.

## 3. Results

### 3.1. Fatty Acid Composition

Table 1 summarizes the data of fatty acid composition, total saturated fatty acids (SFA), monounsaturated (MUFA) and polyunsaturated fatty acids (PUFA) of *Opuntia ficus-indica* seed oil. The five major fatty acids were linolenic acid (C18:3), oleic acid (C18:1) linoleic acid (ω 6; C18:2) followed by palmitic acid (C16:0) and stearic acid (C18:0), representing, respectively 50.69, 21.10, 14.00, 6.73 and 5.74%. Minimal quantities of myristic (C14:0), margaric (C17:0), palmitoleic (C16:1), Arachidic (C20:0), Eicosanoic (Gondoic) (C20:1), Behenic (C22:0), and Erucic (22:1) fatty acids were also identified and quantified. Polyunsaturated fatty acids were the major group of fatty acids, representing 64.69, followed by monounsaturated fatty acids 22.30% and saturated fatty acids 12.98%. The ratio of saturated/unsaturated acid of *Opuntia ficus-indica* seed oil was 0.1, which is low due to the high quantity of unsaturated fatty acid such as C18:3n9, C18:1n9 and C18:2n9.

### 3.2. Tocopherol Content

Table 2 revealed that *Opuntia ficus-indica* seed oil has a high tocopherol profile, which consists of α-, γ- and β-tocopherols. These results showed that total tocopherol was found to be 24.02 μg/mL, where β-tocopherol was found to be the main form of tocopherols in OFI oil scored 42.21%, γ-tocopherol scored 41.13%, and α-tocopherol scored 16.65%.

### 3.3. GC-MS Analysis of Opuntia ficus-indica Seed Oil

**Figure 1:**
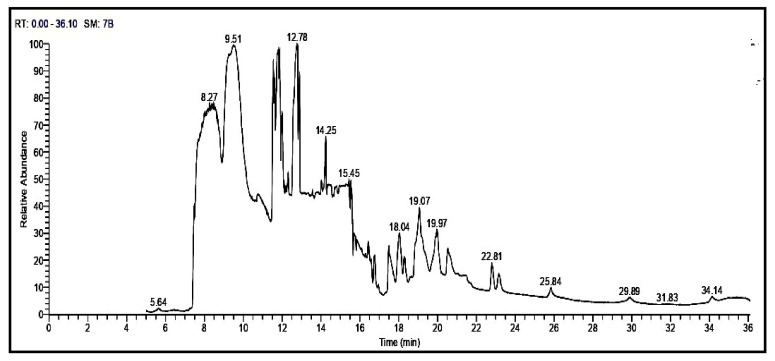
GC-MS analysis of *Opuntia ficus-indica* seed oil.

Totally 21 components were identified through GC-MS according to their peak area and retention time as shown in Table 3 and Figure 1. The studied oil contain different percentages of the major phytosterols, β & γ -sitosterol, Stigmasterol, Campesterol, Stigmast-5-en-3 ol, (3á,24S), which represents (1.67, 2.05,1.36, 4.15, 2.34%) for OFI oil. Furthermore, other constituents detected in the OFI oil in different percentages, such as alcohol triterpenic (9,19-Cyclolanost-24-en-3-ol,acetate, (3á)) or Cycloartenol (3.24%). Esters of fatty acid were found, such as Hexadecanoic acid, methyl ester (6.95%), 9,12,15-Octadecatrienoic acid, methyl ester, (Z,Z,Z)- (5.52%), 7,10,13-Eicosatrienoic acid, methyl ester (7.52%), 9-Octadecenoic acid, 12-hydroxy-, methyl ester, [R-(Z)]- (6.88%), 6,9,12-Octadecatrienoic acid, methyl ester (0.88%), 13-Docosenoic acid, methyl ester (15.10%), Docosanoic acid, methyl ester (0.89%), Eicosanoic acid, methyl ester (4.00%), Tetracosanoic acid, methyl ester (2.19%), Hexacosanoic acid, methyl ester (1.58%). Aromatic compounds (phenolics) were found, such as 1H-Purin-6-amine, (2fluorophenyl)methyl)- (4.75%), (flavonoids) such as 3′,4′,7-Trimethylquercetin (1.77%), 6,8-di-c-á-glucosylluteolin (1.37%).

### 3.4. Total Phenolic Contents

Our study revealed that OFI oil has a total phenolic content of 30.5 mg Gallic acid equivalents (GAE)/mL oil.

### 3.5. Scavenging Activity against DPPH

The scavenging activity of OFI oil versus free radical DPPH recorded a high inhibition rate of 89.2 % at 200 µL/mL of oil (IC_50_ value: 42.12 μg/mL) as compared to beta hydroxy butyrate BHT (IC_50_ = 57.10 µg/mL) as reference scored 80.40%.

### 3.6. Antimicrobial Activity

**Figure 2:**
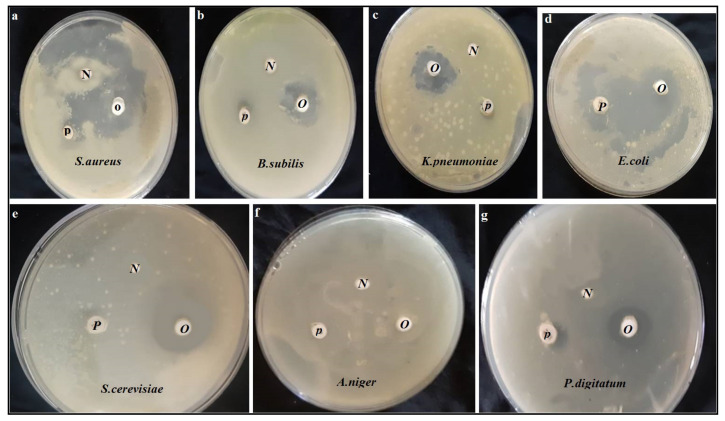
The antimicrobial potency of the OFI oil against tested microbial strains. OFI oil (**O**), negative control (**N**), positive control (**P**).

Table 4 and Figure 2 summarize the antimicrobial potency of the OFI oil using the disc diffusion method. OFI oil was able to inhibit all tested strains with varying diameter from 9.4 to 28.4 mm, except *Aspergillus niger* which showed greater resistance to OFI oil without zone of inhibition (Table 4). The least efficacy of OFI oil against *Pen.digitatum* (fungus) was recorded by region (9.4 ± 0.5), while the most sensitive strain was *Saccharomyces cerevisiae* with a diameter (28.3 ± 0.4mm) compared to (9.2 ± 0.6mm) of Oxytetracycline as a positive control. Diversity of antibacterial power in OFI oil; *E. coli* as Gram-negative bacteria was more sensitive than the *S. aureus* as Gram-positive bacteria by area of inhibition (21.2 ± 0.2mm) and (17.3 ± 0.4mm) respectively. MIC and MBC value were determined to generate the specific dose and nature activity of the OFI oil for use as bacteriostatic or bactericidal. MIC and MBC values in the OFI oil differed according to resistance of microbial strains examined. The lowest MIC and MBC were recorded at 10, 15 µg/mL and 15, 20 µg/mL OFI oil against *Saccharomyces cerevisiae* and *E.coli* respectively.

### 3.7. Cytotoxic Activity

**Figure 3:**
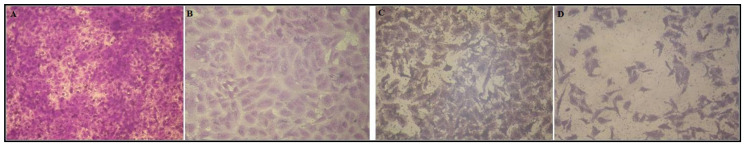
Microscopic observation of the cytopathic effects (morphological alterations) of the prostate carcinoma cells (PC3) treated with *Opuntia ficus-indica* seed oil concentrations (**A**) Control; (**B**) 20 µg/mL; (**C**) 100 µg/mL; (**D**) 500 µg/mL. Figure is at 100× magnification.

Results revealed that OFI oil exhibited highest cell viability (99.63 ± 0.45%) at 250 µg/mL of oil against Mammalian cells from African green monkey kidney (Vero) cells. In case of A-2780 (Ovarian carcinoma) cell line, OFI oil showed weak inhibitory activity (0.72 ± 0.64%, 15.31 ± 1.25%) at 250,500 µg/mL of Oil respectively. Although in the case of PC-3 (Prostate carcinoma) cell line, OFI oil showed significant inhibitory activity (69.33 ± 2.19%, 83.06 ± 1.78%) at 250,500 µg/mL of oil respectively with IC_50_ = 110.28 ± 2.16 µg/mL. Figure 3 represented the microscopic observation of the prostate carcinoma cells (PC3) treated with different concentrations of *Opuntia ficus-indica* seed oil.

### 3.8. Antiviral Activity

Results revealed that OFI oil exhibited low viral inhibition rate (Antiviral effect %) (22.67 ± 2.79%) at 300 µg/mL of oil against herpes simplex type 2 (HSV-2) virus as compared with Acyclovir (93.70 ± 1.19%) as a positive control at 20 µg/mL of it.

## 4. Discussion

The fatty acid composition study of the seed oil of *O. ficus-indica* has shown that this oil belongs to the class of “polyunsaturated” oils [12]. Our results are in agreement with those published by [32] stated that linolenic acid represents the major component of fatty acids in the oil of total lipids of *O. dillenii*, and also the study by [33] where they reported that *O. ficus-indica* oil contains 20.19% of oleic acid, 12.24% of palmitic acid and 3.69% of stearic acid. Also, other fatty acids were identified in minimal quantities in this oil: palmitoleic, myristic, arachidic and behenic. On the contrary, [33] reported that linoleic acid represents the main fatty acid. Furthermore; the fatty acid composition of cactus grown in various regions is significantly different. It is known that this composition is strongly affected by the climatic factors and the type of soil in which it was grown [34].

The high level of total tocopherols is the peculiarity of cactus seed oils [35,36,37]. Our data are concordant with that previously published by [35,37,38] reported that *O. ficus-indica* oil is very rich in tocopherols, generally *β*-tocopherol, *γ*-tocopherol, *α*-tocopherol. Tocopherols, also called Vitamin E, are an important family of lipophilic compounds which have antioxidant activity where the interest is determining the tocopherols composition in *O. ficus-indica* seed oil. Our results concluded that the O. ficus-indica oil is rich in tocopherols (24.02 μg/mL). Thus, high content of vitamin E, seen in oils, may contribute to significant oxidative stability [39].

Our study revealed that the *O. ficus-indica* seed oil contains phytosterols, esterified fatty acids and organic acids with varied percentage. Our data are concordant with that previously published by [3,11,37,38,39,40], which stated that *O. ficus-indica* seed oil is the richest in sterol constituent, compared to oils from other Opuntia species. The next main sterol component of Opuntia oil was campesterol, which is effective for the inhibition of proinflammatory cytokines [41] and inducing cell cycle arrest and prostaglandin release in response to the increased ROS level [42].

The radical scavenging activity of DPPH is one of the accreditation methods for investigating the antioxidant activity of plant extracts [43]. The scavenging activity against free radical DPPH of OFI oil scored a high inhibition rate of 89.2 % at 200 µL/mL of oil as compared to butylated hydroxytoluene (BHT) as reference scored 80.40%. While the total phenolic content was 30.5 mg gallic acid equivalents (GAE)/mL oil, this was in the same line with [44]. The scavenging activity and phenolic components increased by increasing the concentration of OFI oil concentration. The higher antioxidant activity observed in OFI may be relative to higher levels of phenolic compounds and other tocopherols, and sterols present in it. The effect of relationship of phenolic composition in the antioxidant capacity is a well-known fact [45]. The antioxidant potential of OFI oil attributed to its bioactive compounds such as flavonoids, polyphenols, chlorophylls, carotenoids, and tocopherols against the harmful effects of free radicals that cause pathophysiological condition such as diabetes, cardiovascular diseases, and degenerative disorders such as dementia and Parkinson’s disease [46,47,48]. The study of [49] showed that phenolic compounds play a role in extending the food’s shelf-life and act as antioxidants in many biological systems. Ref [50] reported that, in vitro, the inhibition of lipid peroxidation attributed to their ability to isolate free radicals and act as metal chelators, which increased by increasing concentration of OFI oil. In this aspect, [51] concluded that there was a significant relationship between phenolic content and DPPH root scavenging in all examined leafy vegetables (*r* = 0.993, *p* < 0.5). They have shown high efficacy in free radical scavengers due to their redox properties, which can play an essential role in the uptake and neutralization of free radicals, and the quenching of single and triple oxygen or decomposition peroxides.

Phytosterols play key roles in many areas such as nutrition (anticancer properties), medicine (therapeutic production steroids), and cosmetics (creams, lipstick). Furthermore, they have been suggested to have anti-inflammatory, antibacterial, antifungal, antioxidant, anti-ulcerative and antitumor activities [52,53,54]. Moreover, our study revealed that the OFI oil contain esterified fatty acids, alcohol triterpenic (Cycloartenol), Propanoic acid, 2-(3-acetoxy-4,4,14-trimethylandrost-8-en-17-yl),6,8-di-c-á-glucosylluteolin, 3′,4′,7Trimethylquercetin, Hexadecanoic acid, methyl ester, 9,12,15-Octadecatrienoic acid, methyl ester, (Z,Z,Z)-which have antioxidant, anti-inflammatory, antibacterial and antifungal, anticancer, hemolytic and 5-alphareductase inhibitor cancer enzyme inhibitors in pharmaceutical, cosmetics, and food industry actions as reported by other studies [55,56,57,58,59,60].

From the result of this paper, *Saccharomyces cerevisiae* showed a high sensitivity to OFI oil. These data are similar to the results observed by [61] that *S.cerevisiae* had area of inhibition (38–40 mm), while *Candida albicans (yeast)* exhibited smaller area of inhibition against oil red: *Opuntia ficus- indica.* Cactus pear seed oil is rich in compounds that lead to have antimicrobial activity; many researchers have come to the similar results [62]. The antimicrobial effect of OFI oil was varied may be due to a variable chemical component of the oil [63]. The present data revealed OFI oil had antibacterial activity against gram negative bacteria that is inconsistent with [61], who demonstrated that antimicrobial effect of OFI oil against Salmonella Typhi as a Gram-negative bacterium was not detected. This paper showed that OFI oil is more effective against Gram-negative bacteria than Gram-positive bacteria, and is incompatible with [64], and the opposite result may be related to less permeable outer membrane [65]. These results indicated that the effect of OFI oil on *Escherichia coli* was in the same line with [64]. They concluded that *Pseudomonas aeruginosa* was more resistant than *Escherichia coli*, due to its outer membrane rich with lipopolysaccharides, which makes it less permeable [66]. Another reason for resistance exclusion systems, is the pumps that extrude antimicrobial compounds from inside the cell before they cause infection [67]. The present study showed that OFI oil had the lowest bacteriostatic concentration (10 µg/mL (MIC)) and bactericidal concentration (15 µg/mL (MBC) on S. cerevisiae). These data are similar to the results observed by [68] that the OFI oil exerts both a bactericidal and bacteriostatic effects against Enterobacter cloacae.

Our study revealed that OFI oil showed weak inhibitory activity against A-2780 (Ovarian carcinoma) cell line, although, in the case of PC-3 (Prostate carcinoma) cell line, OFI oil showed significant inhibitory activity. These data are similar to the results of [69], who investigated the in vitro chemoprevention effect of prickly pear seed oil at various concentrations (0.01, 0.1, 1, 10, 100 M) versus the growth of HepG2 and Colo-205 cells. On contrary, ref [70] stated that the prickly pear seed extracts taken from different cultivars of prickly pear showed no toxicity to colon, prostate or breast cancer cells in the concentration range of 0.2–0.16 g/mL estimated by the MTT assay. Many in vitro studies have shown that PUFAs have growth-inhibitory and pro-apoptotic effects on various kinds of cancer cell lines [71]. For this reason, the inhibitory effect of OFI oil is due to the high content of PUFAs (linolenic acid and linoleic acid), which are compounds known for their anticancer effect in cancer cells. The α-tocopherol is a predominant component of *Opuntia ficus-indica* oils accounting for 56 mg/kg in oil. The potential health effects of α-tocopherol are powerful as antioxidant effects and the active form of vitamin E that protects the body from cardiovascular and cancer disease. As γ-tocopherol is more powerful than α-tocopherol in preventing prostate cancer cell growth, reducing oxidative DNA damage, scavenging complex and mutagenic and nitrifying oxidative stress [34,72,73,74]. These data are similar to the results of [75], who concluded that OFI oil may have anti-cancer therapeutic effects against colon cancer and adenocarcinoma cell lines. This effect could be elucidated by inducing programmed cell death (apoptosis). OFI seed oil is rich with unsaturated fatty acids (USFA) such as oleic acid (omega-9) plus to β-sitosterol, which led to reducing prostaglandin concentrations (PGE2), and myeloperoxidase activity (MPO) in the inflamed tissues. It has an anti-inflammatory effect [76]. The interest in plant materials containing phenolic compounds is increasing due to their high antioxidant efficacy, which may provide protection against cancer by inhibiting oxidative damage, known to be a possible cause of mutation [77].

Our results revealed that OFI oil exhibited low viral inhibition rate against herpes simplex type 2 (HSV-2) virus in the same line of the only report on antiviral activity of the Opuntia genus is by [78], who found an antiviral effect of the crude extract of *Opuntia streptacantha Lem.* against some viruses of human, horses and mice in cell culture.

## 5. Conclusions

Our data concluded that the cold pressing oil of *Opuntia ficus-indica*, produces interesting bioactive compounds such as fatty acids, tocopherols, sterols, flavonoids and polyphenols, as well as its main biological potentials such as, antioxidant, antiviral, antifungal, antibacterial and anticancer potentials, making it a promising candidate for the application in pharmacology and cosmetics industry.

## Figures and Tables

**Table 1:** Fatty acids percentage of *Opuntia ficus-indica* seed oil.

Fatty Acids	%
**Saturated fatty acids**	
Myristic acid C14:0	0.05
Palmitic acid C16:0	6.73
Margaric acid C17:0	0.06
Stearic acid C18:0	5.74
Arachidic acid C20:0	0.23
Behenic acid C22:0	0.17
**Monounsaturated fatty acids**	
Palmitoleic acid C16:1	0.10
Oleic acid C18:1	21.10
Eicosanoic acid (Gondoic acid) C20:1	0.34
Erucic acid C22:1	0.76
**Polyunsaturated fatty acids**	
Linoleic acid C18:2	14.00
Linolenic acid C18:3	50.69
Σ SFA	12.98
Σ MUFA	22.30
Σ PUFA	64.69

**Table 2:** Tocopherol content of *Opuntia ficus-indica* seed oil.

Compound	RT	Concentration μg/mL
A-Tocopherol	5.0	4.00
γ-Tocopherol	7.0	9.88
Β-Tocopherol	11.0	10.14
Total tocopherols	-	24.02

**Table 3:** GC-MS analysis of *Opuntia ficus-indica* seed oil.

Compound Name	RT	Area %	Molecular Formula	Molecular Weight
Hexadecanoic acid, methyl ester (Palmitic acid, methyl ester)	7.59	6.95	C17H34O2	270
9,12,15-Octadecatrienoic acid, methyl ester, (Z,Z,Z)- (Linolenic acid, methyl ester)	9.13 9.65	5.52 8.97	C19H32O2	292
7,10,13-Eicosatrienoic acid, methyl ester (Methyl eicosa-7,10,13-trienoate)	11.55	7.52	C21H36O2	320
9-Octadecenoic acid, 12-hydroxy-, methyl ester, [R-(Z)]- (Methyl ricinoleate)	11.81	6.88	C19H36O3	312
Eicosanoic acid, methyl ester (Arachidic acid—methyl ester)	11.87	4.00	C21H42O2	326
Stigmast-5-en-3 ol,(3á,24S)-	12.01	2.34	C29H50O	414
6,9,12-Octadecatrienoic acid, methyl ester	12.31	0.88	C19H32O2	292
13-Docosenoic acid, methyl ester	12.74	15.10	C23H44O2	352
Tetracosanoic acid, methyl ester	14.25	2.19	C25H50O2	382
Docosanoic acid, methyl ester (Behenic acid, methyl ester)	15.53	0.89	C23H46O2	354
Hexacosanoic acid, methyl ester	15.59	1.58	C27H54O2	410
6,8-di-c-á-glucosylluteolin	16.45	1.37	C27H30O16	610
á-Sitosterol	16.76	1.67	C29H50O	414
1H-Purin-6-amine, (2fluorophenyl)methyl)-	17.49	4.75	C12H10FN5	243
Campesterol	18.04	4.15	C28H48O	400
Stigmasterol	18.30	1.36	C29H48O	412
ç-Sitosterol	19.07	2.05	C29H50O	414
9,19-Cyclolanost-24-en-3-ol, (3á)-(Cycloartenol)	19.98	3.24	C30H50O	426
3′,4′,7Trimethylquercetin	22.80	1.77	C18H16O7	344

**Table 4:** Measurement of inhibition zone diameter, Minimum Inhibition Concentration MIC, and Minimum Bactericidal Concentration of *Opuntia ficus-indica* seed oil.

Microbial Species	Zone of Inhibition (mm, Mean ± SEM)	MIC (µg/mL)	MBC (µg/mL)	* Oxytetracycline 30 mg	* Penicillin 10 mg
*S. aureus*	17.3± 0.4	20	25	ND	_____
*B. subtilis*	14.4 ± 0.5	25	35	5.1 ± 0.5	_____
*E. coli*	21.2 ± 0.2	15	20	17.0 ± 0.3	_____
*K.pneumoniae*	18.3 ± 0.4	20	25	ND	_____
*S. cerevisiae*	28.3 ± 0.4	10	15	9.2 ± 0.6	_____
*Asp.niger*	ND	ND	ND	ND	ND
*Pen.digitatum*	9.4 ± 0.5	35	40	_____	4.4 ± 0.5

* (OT30) and (P10) were used as positive controls: ND; Not detected.

## Data Availability

Not applicable.

## Sample Availability

Samples of the compounds are available from the authors.
